# Plasma Biology

**DOI:** 10.3390/ijms22115441

**Published:** 2021-05-21

**Authors:** Akikazu Sakudo, Yoshihito Yagyu

**Affiliations:** 1 School of Veterinary Medicine, Okayama University of Science, Imabari, Ehime 794-8555, Japan; 2 Department of Electrical and Electric Engineering, Sasebo National College of Technology, Sasebo, Nagasaki 857-1193, Japan; yyagyu@sasebo.ac.jp

It is now more than 90 years since Irving Langmuir used the technical term “plasma” to describe an ionized gas [1]. Plasma technology has recently expanded to encompass more and more of our daily lives [2]. For example, plasma disinfection/sterilization contributes to public health in the field of medicine and dentistry. Moreover, advances in plasma technology are being exploited to accelerate wound healing, as well as for the development of novel forms of tumor treatment. In the agricultural sector, plasma technology could contribute to higher crop yields by enhancing seed germination and the growth of plants, as well as food preservation and disinfection. Plasma technology could also be utilized in environmental applications, including water treatment/remediation and the treatment of exhaust gases. As such, plasma will be a supportive technology in the provision of clean energy to help achieve sustainable development goals (SDGs). Indeed, the broad potential of plasma technology is only just being realized. However, a large potion of plasma’s the mechanisms of action in biological applications remains unclear.

On the basis of the background outlined above, this Special Issue entitled, “Plasma Biology”, of *International Journal of Molecular Sciences,* includes 14 original articles and 3 reviews providing new insights into the application and molecular mechanisms of plasma biology.

The solutions treated with plasma are referred to as “plasma-activated medium (PAM)” [3], plasma-treated medium (PTM)” [4], “plasma-activated liquid (PAL)” [5], or “plasma-treated liquid (PTL)” [6]. These solutions contain reactive chemical species with a short half-life (e.g., reactive oxygen species (ROS) and reactive nitrogen species (RNS)) that can act as disinfectants [2] or anticancer agents [2].

Hwang et al. [3] examined the efficacy of PAM and tumor necrosis factor-related apoptosis-inducing ligand (TRAIL) in combination (PAM/TRAIL) as an anticancer therapy. PAM/TRAIL showed synergistic effects on growth inhibition in TRAIL-resistant cancer cells via enhanced apoptosis. The antioxidant *N*-acetylcysteine was found to prevent PAM/TRAIL-induced cancer cell apoptosis, suggesting that ROS is related to the induction of PAM/TRAIL-mediated apoptosis.

Terefinko et al. [4] demonstrated PTM, produced by a cold atmospheric pressure plasma (CAP)-based reaction-discharge system, induces apoptosis in cancer cells, especially metastatic cells. 

Dzimitrowicz et al. [5] reported the use of PAL, produced by a direct current atmospheric pressure glow discharge that is generated in contact with a flowing liquid cathode (FLC-dc-APGD). PAL was shown to display antibacterial action against *Dickeya solani* and *Pectobacterium atrosepticum*, which are important plant pathogens. Moreover, the mechanism of action of PAL was possibly due to the presence of ROS and RNS.

Bengtson and Bogaerts [6] developed a mathematical model to investigate the key chemical species involved in the cellular response to PTL, especially hydrogen peroxide (H_2_O_2_). The model can be used to quantify the selective and synergistic anticancer effect of PTL in both susceptible and resistant cells.

In a report by Bekeschus et al. [7], human monocyte-derived dendritic cells (moDCs) were subjected to plasma treatment. Markers, such as CD25, CD40, and CD83, were shown to be activated by this treatment, which is crucial for T cell co-stimulation. Moreover, the plasma treatment increased cytokine levels such as interleukin (IL)-1α, IL-6, and IL-23. These findings suggest that plasma treatment augments costimulatory ligand and cytokine expression in human moDCs.

Zimmermann et al. [8] demonstrated that treatment with a MiniFlatPlaster CAP device induced elevated levels of nitrite and nitrate as well as enhanced acidification. These results highlight the impact of acidified nitrite on melanoma cells and confirm the importance of RNS during CAP treatment. 

Tyczkowska-Sieroń et al. [9] treated *Candida albicans* with a sublethal dose of CAP. Subsequent analysis of the CAP treated yeast identified six single-nucleotide variants, six insertions, and five deletions, as well as the decreased or increased activity of the corresponding enzymes.

Haralambiev et al. [10] examined the use of two different CAP devices, kINPen and MiniJet. A human endothelial cell line (HDMEC) was treated directly and indirectly with CAP. Direct CAP treatment of HDMEC resulted in robust growth-inhibition, whereas indirect CAP treatment did not. Both the migration and tube formation of HDMEC were significantly inhibited after CAP-treatment. In addition, both CAP devices induced HDMEC apoptosis.

Sakudo et al. [11] investigated whether antibiotic-resistant and non-resistant bacteria display differential susceptibility to treatment with plasma [12,13]. *Escherichia coli,* with or without a plasmid that includes an ampicillin resistance gene and chloramphenicol acetyltransferase (CAT) gene, were treated with a dielectric barrier discharge (DBD) plasma torch. The plasma treatment was found to degrade the lipopolysaccharide (LPS) and DNA of the bacteria as well as CAT. Furthermore, the plasma treatment was equally effective against antibiotic-resistant and non-resistant bacteria. This finding suggests that plasma treatment is effective against bacterial strains resistant to conventional antibiotic therapy.

Kwon et al. studied the attachment of human mesenchymal stem cells (hMSCs) to the surface of titanium modified by plasma treatment generated using either nitrogen (N-P), air (A-P), or humidified ammonia (NA-P) [14]. N-P, A-P, and NA-P plasma treatment resulted in a surface with increased hydrophilicity, which promoted enhanced cell attachment compared with the untreated control (C-P). Furthermore, greater cell proliferation was observed on surfaces treated with A-P or NA-P than with C-P or N-P. In addition, the NA-P treated surface resulted in a higher level of alkaline phosphatase activity and osteocalcin expression than the other samples.

Przekora et al. [15] produced silica nanoparticles (NPs) and FexOy/NPs, which are formed of silica NPs decorated with iron oxide (FexOy to denote magnetite + maghemite phase) and embedded them in the polysaccharide matrix of chitosan/curdlan/hydroxyapatite biomaterial. The combined action of CAP and the materials produced on the proliferation and osteogenic differentiation of human adipose tissue-derived mesenchymal stem cells (ADSCs) was then studied. Plasma activation of FexOy/NPs-loaded biomaterial promoted the formation of ROS, resulting in an enhancement of stem cell proliferation without inhibition of osteogenic differentiation.

Han et al. [16] performed large-scale image analysis to investigate the effect of treatment with a plasma jet generating CAP on the cell-cycle stage and quantify damage to nuclear DNA in single cells. S phase cells were found to be more susceptible to DNA damage by the plasma jet treatment than either G1 or G2 phase cells.

Adhikari et al. [17] examined the co-effect of CAP and silymarin nanoemulsion (SN) treatment on the autophagy pathway in a human melanoma cell line. The results showed that CAP and SN together induced autophagy via the phosphoinositide 3-kinase/mechanistic target of rapamycin (PI3K/mTOR) and epidermal growth factor receptor (EGFR) pathways. Moreover, plasma treatment modulated the expression of transcription factors (ZKSCAN3, TFEB, FOXO1, CRTC2, and CREBBP) and specific genes (BECN-1, AMBRA-1, MAP1LC3A, and SQSTM) related to the induction of autophagy.

Sun et al. [18] used high-resolution liquid chromatography mass spectrometry/mass spectrometry (HR-LC-MS/MS) to investigate the structural properties and immunoreactivity of celiac-toxic peptides and wheat storage proteins modified by a plasma jet. The results indicated that plasma jet treatment reduced and modified celiac-toxic peptides by backbone cleavage of QQPFP and PQPQLPY at specific proline and glutamine residues, followed by hydroxylation at the aromatic ring of phenylalanine and tyrosine residues. The immunoreactivity of gliadin extract was reduced by the plasma jet treatment. These observations suggest that the plasma jet could initiate the depolymerization of gluten polymer.

A review by Zubor et al. [19] focuses on the potential of CAP for the management of vulvar cancer. Although no reports have been published concerning the effect of CAP on vulvar cancer cells, progress has been made in gynaecological oncology and in other types of cancer. The review highlights an understudied area that should be a focus for future research.

A review by Chokradjaroen et al. [20] provides insight into solution plasma in aqueous solutions and the potential application of this technology in modifying chitin and chitosan, including degradation and deacetylation.

A review by Braný et al. [21] gives an overview of the current status and future perspectives of CAP in the field of modern medicine. This review article is highly cited and received 22 citations as of 17 May 2021 according to Crossref.

Finally, the Editors are delighted to have had the honor of organizing this Special Issue for *International Journal of Molecular Sciences*, which highlights the research of eminent scientists in the field of plasma biology. The Editors would like to thank all the contributors to this Special Issue for their commitment and enthusiasm during the compilation of the respective articles. The Editors also wish to thank Kaitlyn Wu and other members of the editorial staff at Multidisciplinary Digital Publishing Institute (MDPI) for their professionalism and dedication. Hopefully, readers will enjoy this Special Issue and be inspired with new ideas for future research.

## Data Availability

Not applicable.
